# A CRISPR-based diagnostic tool to survey drug resistance in human African trypanosomiasis

**DOI:** 10.1128/aac.00933-25

**Published:** 2025-11-18

**Authors:** Elena Pérez Antón, Annick Dujeancourt-Henry, Brice Rotureau, Lucy Glover

**Affiliations:** 1 Trypanosome Molecular Biology Unit, Institut Pasteur, Université Paris Cité, INSERM U1347 555089, Paris, France; 2 Trypanosome Transmission Group, Trypanosome Cell Biology Unit, INSERM U1347, Institut Pasteur, Université Paris Cité 555089, Paris, France; 3 Parasitology Unit, Institut Pasteur of Guinea 673901 , Conakry, Guinea; The Children's Hospital of Philadelphia, Philadelphia, Pennsylvania, USA

**Keywords:** human African trypanosomiasis, *Trypanosoma brucei gambiense*, SHERLOCK, crRNA, Cas13a, drug resistance, acoziborole, melarsoprol, pentamidine

## Abstract

The World Health Organization aims to eliminate human African trypanosomiasis caused by *Trypanosoma brucei gambiense* (gHAT) by 2030. With the decline of reported cases, maintaining active surveillance is essential, including for the potential emergence of drug-resistant parasites. We have developed new highly specific diagnostic tools, using the Cas13a-based Specific High-Sensitivity Reporter Enzymatic UnLOCKing (SHERLOCK) technology, for the detection of drug-resistant genotypes that (i) are already circulating, such as the AQP2/3_(814)_ chimera providing resistance to pentamidine and melarsoprol or (ii) could emerge, such as the *Tb*CPSF3 (N^232^H) mutation, associated with acoziborole resistance under laboratory conditions. The *AQP2/3_(814)_* SHERLOCK assay detected RNA from both cultured parasites and field strains isolated from gHAT patients who relapsed following melarsoprol or pentamidine treatment. The *CPSF3_(SNV)_* SHERLOCK assay discriminated between wild-type *CPSF3* RNA and *CPSF3* bearing a single A-C mutation that confers resistance to acoziborole *in vitro*. These SHERLOCK assays are amenable for use as a high-throughput screening method to monitor for drug-resistant-associated mutations in *Trypanosoma brucei*, providing a new molecular tool for epidemiological surveillance during the gHAT elimination phase.

## INTRODUCTION

Human African trypanosomiasis (HAT), or sleeping sickness, is one of the 21 conditions identified as a neglected tropical disease by the World Health Organization (WHO) (1). HAT is caused by an infection with the extracellular protozoan parasite *Trypanosoma brucei (Tb) gambiense* (gHAT) in West and Central Africa or *T. b. rhodesiense* (rHAT) in East and Southern Africa. Both HAT infections follow a typical clinical pattern. Stage 1 is an infection of the blood and lymph, frequently associated with intermittent fevers and lymphadenopathies, and advancing to stage 2 when the parasites invade the central nervous system, resulting in severe neurological symptoms, and ultimately death if untreated. The major difference in disease progression between *T. b. gambiense* and *T. b. rhodesiense* infections is that the former is chronic, lasting over several months to years, and the latter is acute, lasting for several weeks to months. There is currently no prophylactic drug available for HAT, and the approved chemotherapies depend on the parasite species and the stage of the disease (1). Hence, accurate diagnosis and staging of the disease are keys for the selection of the appropriate drug treatment, but diagnosis is based on a tedious algorithm, including lumbar puncture as a confirmatory test for the most advanced stage of gHAT (2).

For gHAT, children under the age of 6 or less than 20 kg are, as a first choice, treated with pentamidine for stage 1 or Nifurtimox-Eflornithine combination therapy (NECT) for stage 2. Patients 6 years and older and more than 20 kg are treated with fexinidazole at stage 1 and at stage 2 (if white blood cell [WBC] count in the cerebrospinal fluid [CSF] is lower than 100 /µL) or with NECT at stage 2 (if WBC in CSF is higher than 100 /µL) (3). For rHAT, children under the age of 6 or less than 20 kg are treated with suramin as a first choice for stage 1 or melarsoprol for stage 2, and if relapse is seen, fexinidazole may be given for compassionate use (3). Patients aged more than 6 years and more than 20 kg are treated with fexinidazole for both stages 1 and 2, yet if relapse is detected, patients are treated with suramin or melarsoprol (3). Both suramin and pentamidine require prolonged intravenous administration, while melarsoprol causes encephalopathic syndrome that is fatal in up to one in ten patients, and NECT involves a long and complex dose regimen with intravenous eflornithine alongside oral nifurtimox uptake over the course of 2 weeks at the hospital (4). Hence, the high toxicity of some of these drugs, especially melarsoprol, as well as the complex administration of NECT, makes access to treatment challenging and has prevented implementation of mass treatment as a method of combating the disease (5).

In addition, drug resistance is not uncommon in the treatment of HAT: resistance to both eflornithine and nifurtimox can be generated *in vitro,* (4) and melarsoprol resistance emerged naturally as early as the 1970s and was widespread by 1990 (6, 7). Pentamidine and melarsoprol share the same mechanism of entry into the parasite cell, through the adenosine transporters and the aquaglyceroporin (AQP) channels (6, 8). Among the three *aquaglyceroporin* genes in *T. brucei* (*TbAQP1-3*) (9), deletion of the *TbAQP2* locus is related to the melarsoprol-pentamidine cross-resistance in bloodstream forms, increasing their effective concentrations (EC_50_) by 2- to 15-fold, respectively (8). Several distinct mutations in the *AQP2-AQP3* genes have been associated with relapse of HAT after treatment with melarsoprol in patients from the Mbuji-Mayi region of the Democratic Republic of the Congo (10, 11) and the Mundri county of South Sudan (10, 12). In certain regions, treatment with melarsoprol has been associated with relapse rates ranging from 20% to more than 50%, highlighting significant concerns regarding treatment failure and potential drug resistance (13–15). In the majority of the relapse cases, a specific chimera containing the first 813 bp from *TbAQP2* and the last 126 bp from *TbAQP3*, here termed AQP2/AQP3_(814)_ (11), was associated with cross-reactivity to melarsoprol and pentamidine (10).

Fexinidazole is currently the only oral drug approved for treatment of both stages in gHAT and rHAT (3, 5) and in children. Fexinidazole was approved in 2019 by the European Medicines Agency (16) as a 10-day oral treatment regimen that must be taken with food for optimal absorption, yet treatment-emergent adverse events, such as vomiting and nausea, increase the risk of non-compliance (5, 17). Due to the high risk of non-compliance leading to the ingestion of suboptimal curative doses, the possibility of relapse is of concern—especially as it may occur up to 24 months post-treatment (5), which could increase the risk of the emergence of drug resistance. Resistance to fexinidazole can be generated under laboratory conditions (18) and occurs through a similar mechanism to that of nifurtimox, that is, via a mutation of the *nitroreductase* (NTR) gene (18). Therefore, prior use of nifurtimox may already have selected for parasites with resistance to fexinidazole (19).

Although not a frontline drug yet, acoziborole promises to be key in the efforts to eliminate HAT. Currently in phase III clinical trials, acoziborole could be approved for the treatment of both stages of gHAT by 2026 (20). It is a single oral dose benzoxaborole derivative that shows high efficacy and safety. Its approval could eliminate the need for routine lumbar puncture and would allow the treatment of parasitology-negative suspects, as well as making treatment more accessible to patients living in remote areas (20, 21). Acoziborole binds to the active site of the Cleavage and Polyadenylation Specificity Factor 3 (CPSF3), which is involved in trypanosome mRNA processing (22), inhibits polypeptide translation and reduces endocytosis of haptoglobin-hemoglobin (23). However, resistance to acoziborole can be generated *in vitro*, through editing a single nucleotide in the *CPSF3* sequence (24).

The CRISPR-based diagnostic—Specific High-sensitivity Enzymatic Reporter unLOCKing (SHERLOCK)—first amplifies nucleic acid using recombinase polymerase amplification (RPA), which is then combined with the Cas13a nuclease, for target recognition via specific guides, and a fluorescent reporter linked to a quencher by nucleotides. Target sequence recognition activates Cas13a’s “collateral effect” of promiscuous ribonuclease activity. So far, SHERLOCK diagnostic assays have been developed for two protozoan parasites, African trypanosomes (25) and *Plasmodium* (26). SHERLOCK is capable of detecting a few attomoles (10^18^ moles) of nucleic acids in a sample and can distinguish between closely related Zika and dengue viruses in clinical isolates, or mutant strains with unique SNPs (27–29). Furthermore, the use of SHERLOCK as a tool for detecting drug-resistant genotypes has been described in the context of *Plasmodium* infection (26) and compared with the current gold standard methods. In a context where the therapeutic arsenal for treating HAT is limited, the emergence of drug resistance is possible where a selective pressure to survive is applied to parasites. This places both existing and potential future drug treatments at risk. To address this, we have developed highly specific SHERLOCK assays that allow detection of specific drug-resistant-associated mutations and that could be essential for epidemiological surveillance in the context of gHAT elimination and rHAT control.

## MATERIALS AND METHODS

### Trypanosome cell lines and isolates

*T. b. brucei* Lister 427 bloodstream form cells (wild type, *in vitro*, Table 1) were cultured in HMI-11 medium with 10% fetal bovine serum (Sigma-Aldrich) at 37°C with 5% CO_2_. RNA extraction was carried out from the culture harvest at 1 × 10^6^ cells/mL. RNA extraction of *T. b. brucei* cells, *T. b. gambiense* ELIANE strain (wild type, *in vitro*, Table 1) cell pellets, and human embryonic kidney (HEK) 293T cells was carried out using the RNeasy Mini kit (Qiagen). Lyophilized RNA extracted from *T. b. brucei* edited CPSF3 (22) (CPFS3 A-C mutant, Table 1, referred to in the text as *CPFS3_(SNV)_*) was resuspended in nuclease-free water. As positive controls for the development of the AQP2/3_(814)_ SHERLOCK assay, we used (i) *T. b. brucei* cells with the *TbAQP2* and *TbAQP3* genes knocked out, expressing the AQP2/3_(814)_ chimera (based on the 40AT field isolated strain sequence, KF564935.1), hereafter referred to as ChAQP2/3 cells or ChAQP2/3_(814)_ RNA (Table 1); (ii) the pRPa-GFP-chimAQP2/3 plasmid (30) containing the same AQP2/3(814) chimera sequence (KF564935.1), hereafter referred to as ChAQP2/3_(814)_ plasmid or ChAQP2/3_(814)_ DNA.

**TABLE 1:** Trypanosome strains used as control for the development of the SHERLOCK assays

Species	Strain	Source	Ref
*T. b. brucei*	Wild type	Strain maintained *in vitro*	
*T. b. brucei*	AQP2/3_(814)_ chimera	Modified *in vitro* cell line	(11)
*T. b. brucei*	CPFS3 A-C mutant	Modified *in vitro* cell line	(22)
*T. b. gambiense*	Wild type	Strain maintained *in vitro*	(31, 32)

### *Lw*Cas13a enzyme expression and purification

The plasmid pC013-Twinstrep-SUMO-huLwCas13a (Addgene plasmid #90097) was used for the protein expression in *Escherichia coli* Rosetta 2(DE3) pLysS-competent cells (28). The protein expression and purification of *Leptotrichia wadei* Cas13a (*Lw*Cas13a) enzyme were performed as described in reference (33) with slight modifications (25). TB medium was reconstituted by adding 47.8 g of TB powder to a 1 L flask, and adding 8 mL of 100% (wt/vol) glycerol. Cell pellet was lysed with supplemented lysis buffer composed of two complete Ultra EDTA-free tablets (Roche), 500 mM NaCl, 100 mg lysozyme, and 125–625 ng deoxyribonuclease I from bovine pancreas (Sigma) to each 100 mL of lysis buffer. The *Lw*Cas13a was stored in single-use aliquots at −80°C to avoid freeze-thaw cycles.

### Design of RPA primers and crRNA

RPA primer pairs were designed using NCBI Primer-BLAST (34) using the custom parameters specified in (33). For the wild-type CPSF3 SHERLOCK, we use the reference sequence XM_839191.1 (Tb927.4.1340). For targeting the chimeric AQP2/AQP3_(814)_, the reference sequence KF564935.1 (*T. b. gambiense* strain 40AT) was used. The RPA primers used in the study are included in Table S1. RPA forward primers include at the 5′ end, the T7 promoter sequence (5′-GAAATTAATACGACTCACTATAGGG-3′) that allows *in vitro* transcription of the amplified target by T7 polymerase.

The DNA templates used to generate the crRNAs consist of (i) the target sequence (5′−3′) which will be the variable region between crRNA, also known as the spacer (with variable lengths from 20 to 28 nt), followed by (ii) the direct repeat template common to all *Lw*Cas13a crRNAs (5′-GTTTTAGTCCCCTTCGTTTTTGGGGTAGTCTAGTCTAAATC-3′), followed by (iii) the T7 promoter sequence at the 3′ end (5′-CCCTATATAGTGAGTCGTATTAATTTC-3′), which will allow *in vitro* transcription of the guides. DNA templates used are listed in Table S1. All oligonucleotides were synthesized by ThermoFisher.

### *In vitro* transcription and purification of crRNAs

The crRNA synthesis was carried out using a DNA template and *in vitro* transcription mediated by T7 polymerase according to the manufacturer’s instructions using the HiScribe T7 Quick High Yield RNA Synthesis Kit (NEB), as previously described (33). Briefly, we first annealed the crRNA DNA template and T7-3G oligonucleotide (5′-GAAATTAATACGACTCACTATAGGG-3′) by denaturation for 5 min, followed by slow cooling (25). After *in vitro* transcription, the crRNAs were purified using magnetic beads (Agencourt RNAClean XP) and stored at 300 ng/µL in single-use aliquots at −80°C to avoid freeze-thaw cycles.

### SHERLOCK assay

The SHERLOCK assay was performed as described in reference (33). The SHERLOCK reaction is a combination of a pre-amplification method by a reverse transcription (RT) recombinase polymerase amplification (RPA) (35) and a specific RNA-target recognition by Cas13-CRISPR RNA-guide (crRNA) machinery (Abudayyeh et al., 2016). The RT-RPA was carried out using the TwistAmp Basic kit (TwistDx) following the manufacturer’s instructions with the addition of the 2.2 U reverse transcriptase Transcriptor (Roche) to enable RT for 45 min at 42°C, with an input volume of 3 µL in a total volume of 11 µL, as described in reference (25). The amplification step is followed by simultaneous *in vitro* transcription of the amplified target by T7 polymerase (Biosearch technology) and detection of the *Lw*Cas13a-crRNA using the same conditions as described in reference (25). For this purpose, 4 µL of the RT-RPA product of each replicate was mixed with the following components in the following concentrations: 20 mM HEPES, pH 6.5, 9 mM MgCl2, 1 mM rNTP mix (NEB), 40 nM of *Lw*Cas13a, 2 U of Murine RNase inhibitor (NEB), 25 U of NxGen T7 RNA Polymerase (Biosearch technology), 25 nM of crRNA and 125 nM of RNaseAlert probe V2 (Invitrogen), in a final volume of 80 µL. The reaction was performed in 384-well plates, F-bottom, μClear bottom (Greiner), incubated at 37°C in a TECAN plate reader (INFINITE F200 PRO M PLEX), in which 20 µL × 3 of each replicate reaction was distributed. Fluorescence measurements were collected every 10 minutes up to 3 hours. All SHERLOCK reactions were performed in triplicate, and three fluorescence measurements were taken from each replicate. A negative control template (NCT) was added in parallel to each independent assay by supplementing nuclease-free water as input. Positive (on-target) and/or negative (off-target) controls were also included in each reaction to ensure the proper performance of the reaction.

### Statistical analysis

The fluorescence intensity values obtained from the TECAN plate reader were analyzed using Excel. For each triplicate reaction at each timepoint, the mean fluorescence intensity value was divided by the mean of the fluorescence intensity value of the NCT at the same timepoint, to obtain the fold-change over background fluorescence. The formula used is as follows:


$$Fold-change\;over\;background\;fluorescence=\frac{Mean\;fluorescence\;intensity\;of\;a\;triplicate\;sample\;reaction\;at\;time\;(x)}{Mean\;fluorescence\;intensity\;of\;the\;triplicate\;NCT\;at\;time\;(x)}$$

The graphs and statistical analysis were performed using GraphPad Prism (version 9.3.1 and 10.4.1) software. The Shapiro-Wilk normality test was performed to evaluate the type of data distribution. The non-parametric Mann-Whitney U test or the parametric bilateral unpaired t-test, with Welch’s correction, according to the data distribution results, was used for the statistical comparison with a 95% confidence interval. For multiple comparisons, we applied the two-stage linear step-up procedure (36). Asterisk represents *P* values at (*) *P* < 0.05, (**) *P* < 0.01 (***), *P* < 0.001, and (****) *P* < 0.0001. Multiple sequence alignments were performed using Clustal Omega (37).

The establishment of a positivity threshold (TH) for the *AQP2/3_(814)_*-specific SHERLOCK assay was assessed using Receiver Operating Characteristic (ROC) curve analysis (GraphPad Prism version 10.4.1) by comparing positive and negative controls (*n* = 142). Data from SHERLOCK assays targeting the *ChAQP2/3_(814)_* RNA (extracted from the ChAQP2/3_(814)_ cells) and DNA (ChAQP2/3_(814)_ plasmid) were categorized as positive controls, while material from non-target parasites and host samples was treated as negative samples for ROC analysis and threshold determination. ROC curves were generated using the Wilson/Brown method with a 95% confidence interval. Thresholds were determined based on Youden’s index (sensitivity + specificity – 1), which identifies the threshold that optimizes classification accuracy (38, 39). For the SHERLOCK assay, the positivity threshold was set according to the ROC-derived value that achieved maximum specificity and sensitivity, 100.0%.

## RESULTS

### Selection of SHERLOCK targets to detect the AQP2/AQP3_(814)_ chimera

**Fig 1:**
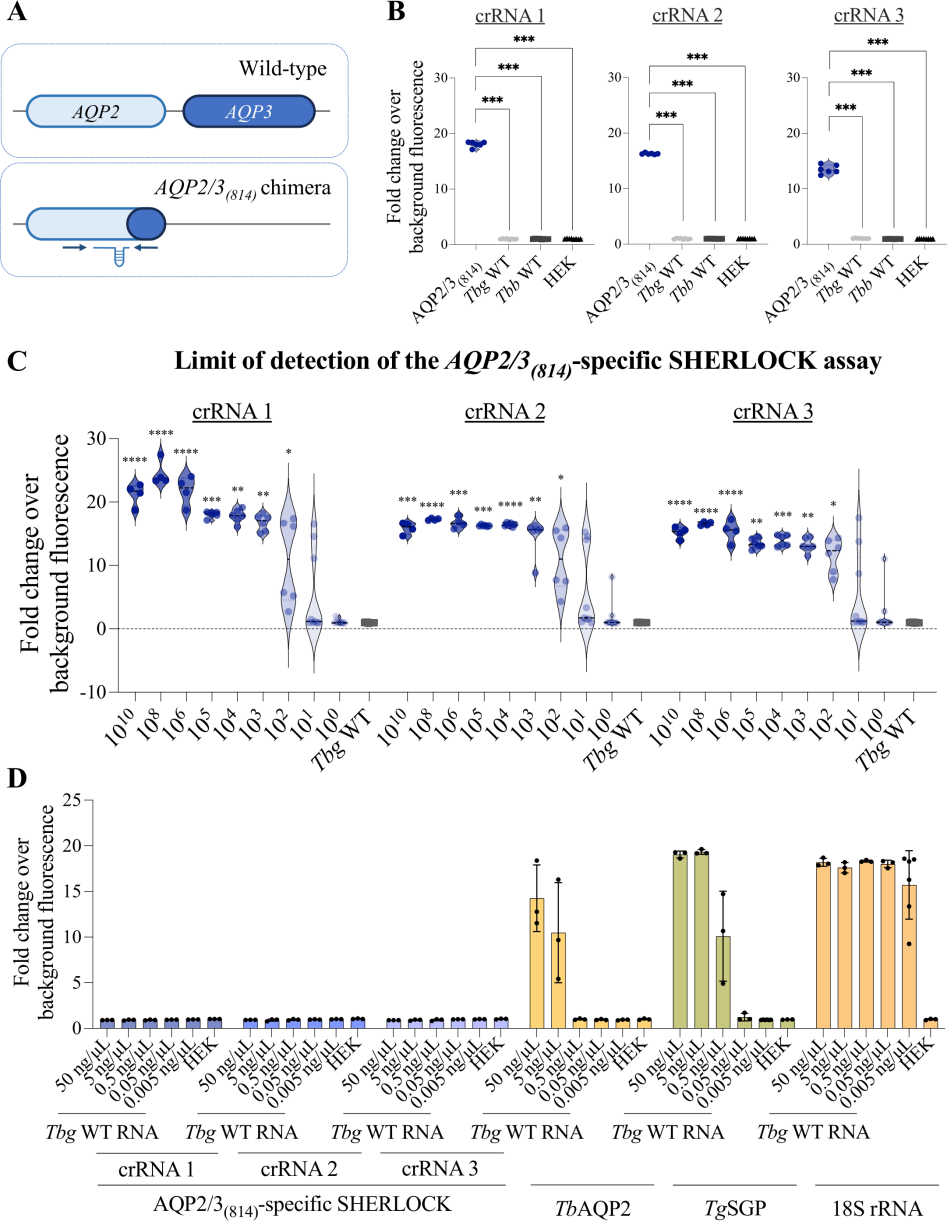
SHERLOCK detection of the *AQP2/3_(814)_* chimera. (**A**) Schematic of the *AQP2* and *AQP3* wild-type locus (top) and the *AQP2/AQP3_(814)_* chimera (bottom). The RPA primers (arrows) and crRNA target (RNA guide) regions for detection of the *AQP2/AQP3_(814)_* chimera are shown. (**B**) crRNA screening for the detection of the *AQP2/AQP3_(814)_* chimera using 10^5^ copies/µL of plasmid containing *AQP2/AQP3_(814)_* sequence (GenBank accession KF564935) as input (four replicates), and RNA (5 ng/µL) from WT *T.b.b*. (*T. b. brucei*), WT *T.b.g*. (*T. b. gambiense*), and human embryonic kidney (HEK) cells (8 replicates of each control). (**C**) Limit of detection for each crRNA using the *AQP2/3_(814)_*-specific SHERLOCK assay. Plasmid containing the chimeric *AQP2/3_(814)_* sequence (DNA) used at concentrations from 10^0^ to 10^10^ c/µL, and RNA from WT *T.b.g*. as a control. The statistical significances shown with asterisks in B and C were derived from the independent comparisons by the Mann-Whitney U test between the readout of the different plasmid concentrations *versus* that of WT *T.b.g* RNA. Asterisk represents *P* values at (*) *P* < 0.05, (**) *P* < 0.01 (***), *P* < 0.001, and (****) *P* < 0.0001. Violin plots, from B and C, represent all the replicates (at least four replicates per condition) evaluated and the median as well as the probability density of the data. (**D**) Screening of increasing amounts of *T.b.g*. WT RNA with the different crRNAs of the *AQP2/3_(814)_*-specific SHERLOCK assay, as compared to the *TbAQP2*-specific, *TgSGP*-specific, and *18S rRNA* pan-*Trypanozoon*-specific SHERLOCK assays. The range of WT *T.b.g*. RNA concentration used was from 50 to 0.005 ng/µL, and RNA from HEK cells (5 ng/µL) was used as a control. The graph displays the mean ± standard deviation (SD), with individual replicate values represented as dots (at least three replicates per condition).

We have adapted our SHERLOCK4HAT (25) workflow for the detection of melarsoprol resistance through the formation of an *AQP2/3* chimera (Fig. 1A). This specific *AQP2/3*_(814)_ chimera has independently arisen in two distinct HAT foci and makes up 31.7% of all known melarsoprol-resistant HAT infections (10, 11). For this assay, we designed the following primers: the RPA forward primer amplifies from nucleotide position 705 of the *TbAQP2* sequence, and the RPA reverse primer from position 841 in the *TbAQP3* sequence (Fig. 1B). We aligned the sequences of the wild type (WT) *TbAQP2, TbAQP3* (Tb927.10.14170, Tb927.10.14160, respectively; Fig. S1) and *AQP2/3*_(814)_ chimera (KF564931.1) to select non-homologous regions between *TbAQP2* and *TbAQP3* genes for primer-binding sites (Fig. 1B). The crRNA guides were designed to target the *TbAQP2* region of the *AQP2/3*_(814)_ chimera (Fig. 1B). We assessed the specificity of the *AQP2/3_(814_*_)_ SHERLOCK assay using the pRPa-GFP-chimAQP2/3 plasmid (30) and RNA extracted from wild-type *T. b. gambiense*, *T. b. brucei,* or human HEK cells (Fig. 1B). HEK cells were used to verify the absence of cross-reactivity with human RNA. All three crRNAs specifically detected the *AQP2/3_(814)_* chimera and did not cross-react with the WT trypanosome or human cell nucleic acids (Fig. 1B). To test the cRNAs on *T. brucei* transcripts, RNA was extracted from *in vitro* derived *TbAQP2* and *TbAQP3*-null cells where the *AQP2/3_(814_*_)_ chimera was expressed from the rRNA intergenic region (30) (Fig. 1C). Again, all three crRNAs selectively detected RNA from cells expressing the *AQP2/3_(814)_* chimera, with crRNA 2 and 3 outperforming crRNA 1 (Fig. S2A).

To ensure the specificity of the AQP2/3_(814)_ SHERLOCK for the chimera, we tested the assay on *T. b. gambiense* WT RNA (Table 1). No signal was detected using any of the three AQP2/3_(814)_-specific SHERLOCK crRNAs (Fig. 1D). Positive detection of WT *T. b. gambiense* RNA was confirmed using SHERLOCK assays specifically designed to detect *TbAQP2* or *TbgSGP* (25), or a pan-*Trypanozoon 18S rRNA* SHERLOCK assay (40) (Fig. 1D; Fig. S1C and S3). Our SHERLOCK AQP2/3_(814)_ assay is specific for the chimera only, even in the presence of the WT *AQP2* gene.

For the application of the AQP2/3_(814)_-specific SHERLOCK assay to the analysis of patient-isolated samples, a threshold (TH) of positivity was established using Receiver Operating Characteristic (ROC) curve analysis. ROC curve analysis evaluates the discriminatory performance of a diagnostic test by plotting sensitivity versus specificity across different decision thresholds. The AQP2/3_(814)_-specific SHERLOCK ROC curve analysis was based on comparisons between more than 58 positive and 84 negative control templates. To reach both a sensitivity and a specificity of 100%, the threshold was set at >1.260 fold change over background fluorescence for crRNA 2 and >1.455 for crRNA 3 (Fig. S4).

### The AQP2/3(814)-SHERLOCK assay reliably identified gHAT relapse cases

**Fig 2:**
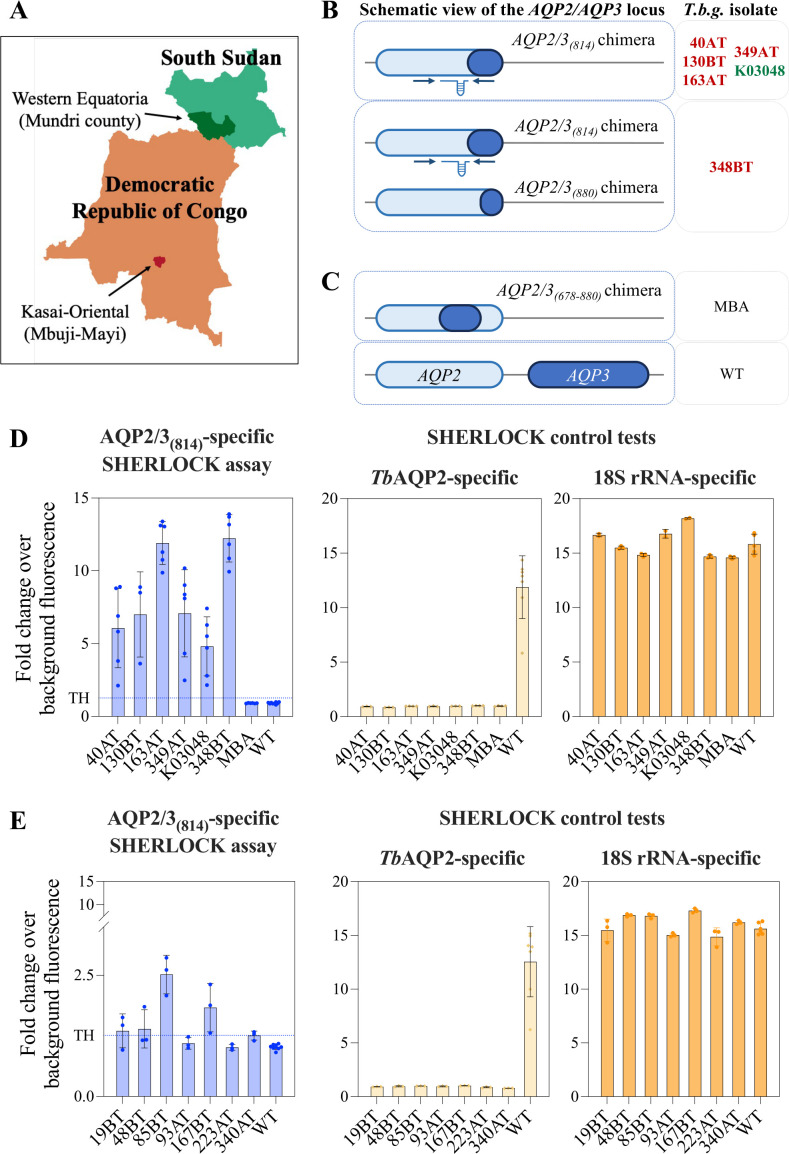
Evaluation of the *AQP2/3_(814)_* SHERLOCK assay on trypanosome isolates with *AQP2/3* chimeras from HAT patients with relapses. (**A**) Map showing the origins of the samples from patients with relapse after treatment. The map was customized with the map generator, MapChart. (**B and C**) Schematics of the *AQP2* and *AQP3* loci in five different strains isolated from patients in the Mbuji-Mayi region, DRC (40AT, 130BT, 163AT, 349AT, and 348BT in red) and the Mundri county, South Sudan (K03048 in green), one strain isolated in Kinshasa, DRC (MBA), and a WT strain. The RPA primers (arrows) and crRNA target (RNA guide) regions for detection of the *AQP2/3_(814)_* chimera are shown. (**D**) Representation of the fold-change over background fluorescence using the *APQ2/3_(814)_*-specific SHERLOCK assay testing the different RNAs extracted from the eight strains isolated from patients: 40AT, 130BT, 163AT, 349AT, 348BT, K03048, MBA, and WT (left graph). The dotted lines show the thresholds of positivity established by ROC analysis for the crRNA 2 used in this study (Fig. S4). The *TbAQP2* and *18S rRNA*-specific SHERLOCK control tests were used in parallel to evaluate the same extracted RNA samples (right graphs). For all replicates, an input concentration of 5 ng/µL was used. (**E**) Representation of the fold-change over background fluorescence using the *APQ2/3_(814)_*-specific SHERLOCK assay (left panel), and the *TbAQP2* and *18S rRNA*-specific control assays (right panels), testing the different RNAs extracted from additional patients in Mbuji-Mayi, DRC (19BT, 48BT, 85BT, 93AT, 167BT, 223AT, and 340AT) and a WT strain. The input concentration used was conditioned by the availability of the sample in the range of 1.5–2.5 ng/µL. The graph represents the mean with SD and shows all replicates with dots. At least three replicates per condition, except for the *18 rRNA* SHERLOCK control assay, where only two replicates were performed for some of the samples due to material limitations.

Two geographically distinct locations have shown high relapse rates following treatment with melarsoprol—the Mbuji-Mayi region of the Democratic Republic of the Congo (DRC) and the Mundri County in South Sudan (Fig. 2A) (13–15). Both are associated with the circulation of parasite strains with mutations and chimerization in the *AQP2/3* genes (10–12) (Fig. 2B). We therefore evaluated whether our AQP2/3_(814)_-specific SHERLOCK assay could discriminate between the clinically relevant chimeric variant—highly prevalent in these regions with elevated relapse rates (Fig. 2B)—without detecting other non-resistant variants, such as the MBA strain bearing the *AQP2/3 _(678–880)_* mutation and isolated from a patient in Kinshasa, DRC, or an *AQPs* wild-type strain isolated from a patient in Côte d'Ivoire (WT) (Fig. 2C; Table 2).

**TABLE 2:** Detailed information on the strains isolated from patients used for AQP2/3_(814)_ SHERLOCK assay validation^*a*^

Strain code	Before inclusion in Pyana et al. (41)		Findings from Pyana et al. (41)		Geographic origin	Date
	Outcome	Treatment	Outcome	Treatment		
40AT			Relapse (6 m)	M10	Mbuji-Mayi, DRC	2006
130BT	Relapse	MN	Probable relapse (24 m)	E14		2006
163AT	Relapse	M3	Relapse (3 m)	MN		2006
349AT			Relapse (3 m)	M10		2006
348BT			Cure	M10		2006
19BT			Cure	E14		2008
48BT			Relapse (6 m)	M10		2008
85BT	Relapse	M3	Cure	E14		2008
93AT			Relapse (6 m)	M10		2006
167BT			Relapse (6 m)	M10		2008
223AT			Relapse (6 m)	M10		2006
340AT			Relapse (3 m)	M10		2006
MBA (11)	Unknown				Kinshasa, DRC	2007
K03048 (10, 12)	Relapse	Melarsoprol			Mundri County, SSD	2003
ELIANE WT (11)	Unknown				Daloa, CI	

^*a*^ DRC: Democratic Republic of Congo; SSD: South Suda; CI: Côte d'Ivoire; BT: before treatment; AT: after treatment; M3: 3 × 3 days melarsoprol treatment; M10: 10 days melarsoprol treatment (2.2 mg/kg/day IV); MN: 14 days melarsoprol (1.8 mg/kg/day IV for 10 days)-nifurtimox (15 mg/kg/day PO for 14 days) treatment; E14: 14 days eflornithine treatment (4 × 100 mg/kg/day IV every 6 h), m, months.

We initially tested 8 samples isolated from gHAT patients and with a genomic characterization of the *TbAQP2/TbAQP3* locus available: (samples 1–5) the 40AT, 130BT, 163AT, 349AT, and K03048 *T. b. gambiense* isolates from patients with relapse and bearing the *AQP2/3_(814)_* chimera (10–12) (Table 2; Fig. 2B); (sample 6) the 348BT strain isolated from a cured patient that contains both *AQP2/3*_(814)_ and *AQP2/3*_(880)_ chimeras (with the first 879 bp from *TbAQP2*, a point mutation at T869C, and the last 60 bp from *TbAQP3*); (sample 7) the *T. b. gambiense* MBA isolate sampled from a patient with unknown treatment outcome and containing an *AQP2*/3 chimera with the first 677 bp from *TbAQP2*, 202 bp of *TbAQP3,* and the last 60 bp from *TbAQP2*, termed as *AQP2/3*_(678–880)_; and (sample 8) a WT *T. b. gambiense* ELIANE strain with no mutation in the *AQP2/3* locus and isolated prior treatment from a patient without relapse (Table 2; Fig. 2C; Fig. S1B) (11).

As expected, the 163AT, 40AT, 349AT, K03048, and 348 BT samples showed a robust signal in the SHERLOCK AQP2/3_(814)_ assay. These results confirmed that the SHERLOCK *AQP2/3*_(814)_ assay can detect the specific mutation associated with melarsoprol resistance from patient samples. As expected, the AQP2/3_(814)_-specific SHERLOCK assay should not detect the *AQP2/3*_(678-880)_ (KM282034), *AQP2/3*_(880)_ (KM282050), or *AQP2/3*_(678-880)_ chimeras (Fig. 2B and C). In parallel, the samples were evaluated using two SHERLOCK assays as controls: WT *TbAQP2* and pan-*Trypanozoon 18S rRNA* assays. As expected, only the WT sample produced a positive signal using the SHERLOCK *TbAQP2* assay, whereas all samples showed a comparable positive signal by the generic pan-*Trypanozoon 18S rRNA* SHERLOCK assay (Fig. 2D).

In addition, we applied our *AQP2/3_(814)_*-specific SHERLOCK assay to a set of seven samples previously analyzed by Pyana et al. (11) using PCR-RFLP that inferred the presence of chimeric *AQP2/3* variants (Fig. 2E). These samples were obtained from patients who experienced relapse after treatment with melarsoprol, except for one case (19BT), in which the patient had not received melarsoprol but was successfully cured following treatment with eflornithine. All samples originated from the same geographic region, known for a high prevalence of drug-resistant strains and chimeric *AQP2/3* variants. Our SHERLOCK assay detected the presence of the *AQP2/3_(814)_* chimera in four of the samples (19BT, 48BT, 85BT, and 167BT; Fig. 2E), including the sample from the patient cured by eflornithine (19BT) (Fig. 2E; Table 2). Although the remaining three samples did not cross the SHERLOCK validation threshold for detecting the *AQP2/3_(814)_* chimera, we hypothesize that this may be due to a possible partial loss of RNA integrity during long-term storage. Furthermore, the SHERLOCK control assay targeting the wild-type *TbAQP2* sequence was negative in all replicates, suggesting the likely complete *AQP2* gene deletion and its total chimerization. Nevertheless, our control SHERLOCK assay targeting *18S rRNA* detected positive signals in all samples, confirming the presence of trypanosome genetic material (Fig. 2E). As the multicopy gene *18S rRNA* is highly expressed, it may be more tolerant of RNA degradation than the single-copy *AQP2/3_(814)_* chimera, making the *18S rRNA* SHERLOCK assay more robust.

### SHERLOCK detection of a single-nucleotide variant associated with acoziborole resistance

Taking advantage of the specificity of SHERLOCK, we developed an assay that could discriminate between the WT and the *in vitro* derived single-nucleotide variant (SNV) of the *CPSF3* gene (*CPSF3_(SNV)_,* accession number XM_839191: N^232^H, AAT-CAT), hereon referred to as *CPSF3_(SNV)_*, which confers resistance to acoziborole (22, 42). We first screened RPA primer pairs that were either 30 nt or 25 nt long (Fig. S5) and designed to amplify the region containing the SNV. The RPA primer pair target amplification was assessed using four different crRNA guides specific for the detection of *CPSF3_(SNV)_*. We observed higher fold-change values with the 30 nt RPA primer pair as compared to the 25 nt primer pair (Fig. S5). Therefore, we selected the long RPA primer pair for subsequent optimization of the SHERLOCK assay.

**Fig 3:**
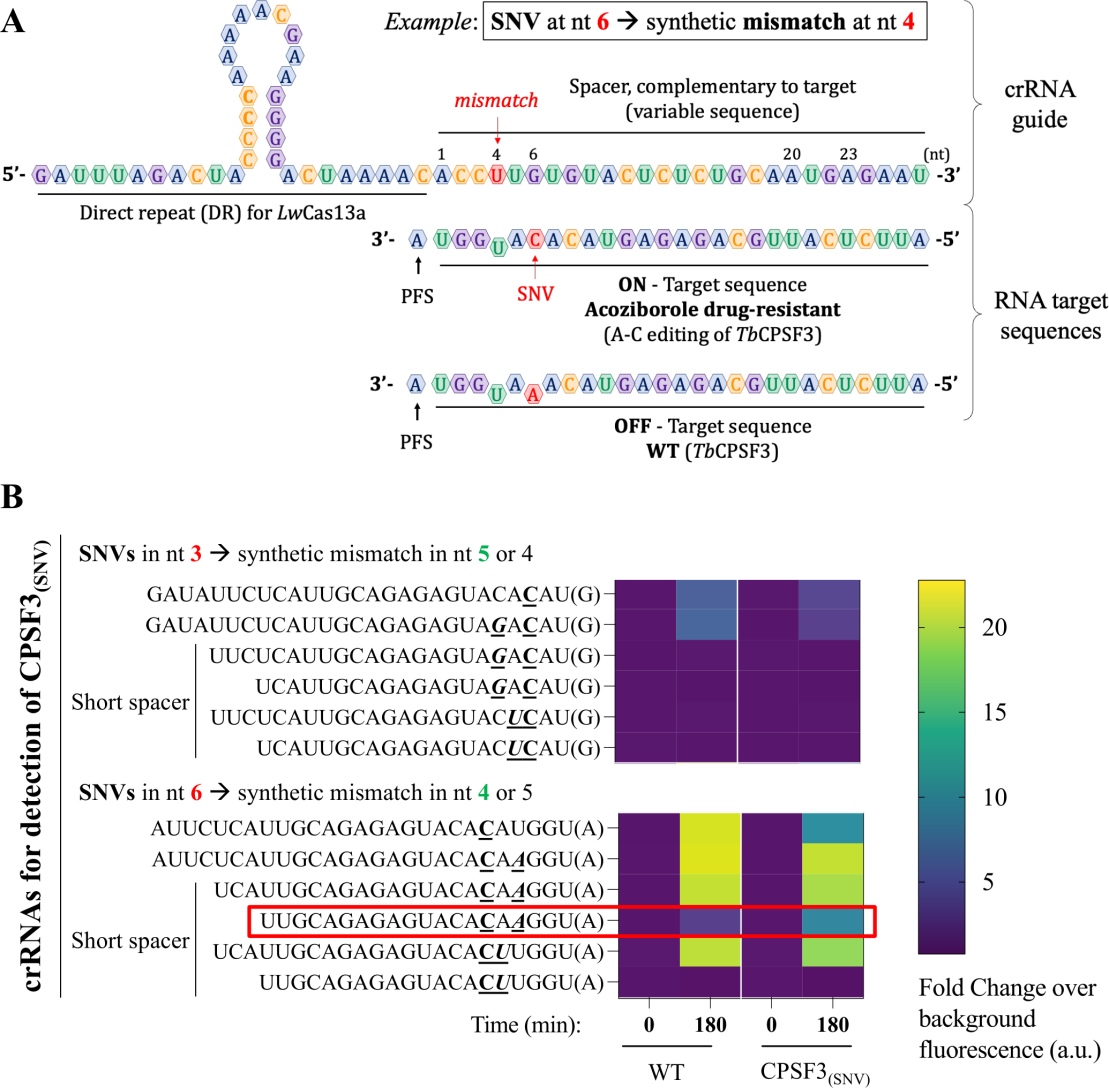
Optimization of a SHERLOCK assay for the detection of *CPSF3* SNV. (**A**) Schematic of the crRNAs design for the detection of the SNV in the *CPSF3* gene. Position of the SNV in red and the inclusion of the synthetic mismatch along the crRNA spacer in green. (**B**) Screening of crRNAs designed for the detection of *CPSF3*-edited cells. The target sequence of each crRNA is indicated, as well as the PFS in brackets. crRNAs used for detection of the *CPSF3_(SNV)_*: (i) SNV fixed at position 3, and (ii) SNV fixed at position 6. SNV is in underlined and bold character, and a synthetic mismatch is in underlined, bold, and italicized character. crRNAs with different spacer lengths were evaluated (20, 23, and 27 nt). The heat map shows the fold-change over background fluorescence intensity values collected at time zero and after 180 minutes of *Lw*Cas13a detection reaction. Three independent replicates were performed for each crRNA, with three fluorescence measurements obtained per replicate. The crRNA with optimal SNV discrimination is highlighted with a red box. G, guanine; A, adenine; U, uridine; C, cytosine; PFS, protospacer flanking site; *Tb*CPSF3, *Trypanosoma brucei gambiense* cleavage and polyadenylation factor 3; WT, wild type.

Following this, 12 crRNA guides were screened for the detection of the *CPSF3_(SNV)_*. We designed crRNAs using the following criteria: (i) setting the SNV complementary base at position 3 (counting from the 3′ end of the crRNA) and the artificial mismatch (if any) at position 5 and (ii) setting the SNV complementary base at position 6 and the mismatch (if any) at position 4 (26, 28, 43) (Fig. 3A). In addition, we designed crRNAs with spacer lengths shorter than described as optimal for *Lw*Cas13a (28 nt in (33). Indeed, reducing the spacer length to a maximum of 20 nt reduced the enzyme activity but maintained, or even improved, its ability to discriminate single mismatches by reducing background fluorescence in the off-target sample (28, 43, 44) (Fig. 3B). Finally, since consecutive double substitutions in the spacer have been described as effective in the loss of collateral cleavage activity of the Cas13a enzyme (44), we also evaluated the efficacy of placing the synthetic mismatch adjacent to our SNV, to generate a double mismatch in the off-target sequence (Fig. 3B).

**Fig 4:**
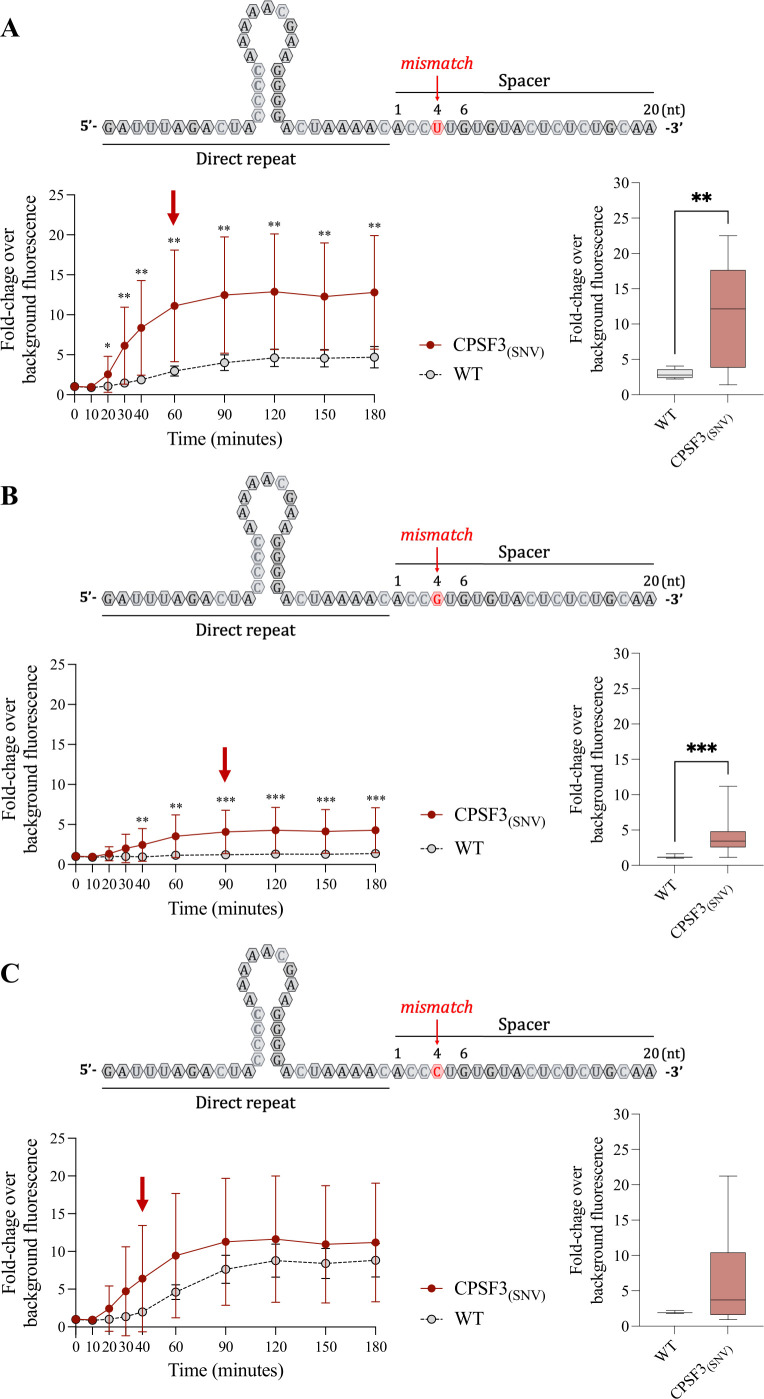
Evaluation of selected nucleotides used as synthetic mismatches in the *CPSF3_(SNV)_* SHERLOCK assay. Kinetics of the *Lw*Cas13a reaction for the selected base with the synthetic mismatch at nt 4: uracil (**A**), guanine (**B**), or cytosine (**C**). The fold-change over background fluorescence is indicated at different time points of the *Lw*Cas13a reaction. Results from WT cells are shown with open circles and those from edited *CPSF3_(SNV)_* cells with red dots. Box plots represent fold-change over background fluorescence intensity values using RNA from WT cells (gray box plots) or RNA from *CPSF3_(SNV)_*-edited cells (red box plots) at the selected time point, here indicated by a red arrow in each kinetic plot. RNA input at 5 ng/µL. Graphs represent data from three independent experiments in panel (**A**), two independent experiments in panel (**B**), and one experiment in panel (**C**). Each experiment included three replicates for the control (WT) and nine replicates for the mutant (CPFS3^mut^) condition. Line graphs show mean ± standard deviation (SD). In box plots, the central line represents the median, and whiskers indicate the minimum and maximum values. Statistical significance was assessed using the Mann-Whitney U test. For multiple comparisons in the kinetic line graphs, the two-stage linear step-up procedure (36) was applied. Asterisks denote significance levels: (*) *P* < 0.05, (**) *P* < 0.01 (***), and *P* < 0.001.

The crRNAs designed to detect the SNV at position 3 showed lower fluorescence emission and were undetectable when using the crRNAs with a spacer length of 20 or 23 nt (Fig. 3B). In contrast, the crRNAs detecting the SNV at position 6 gave high fluorescence intensity values with both WT and *CPSF3_(SNV)_*. The fluorescence was reduced only when the length of the spacer was limited to 20 nt (Fig. 3B). The best-performing crRNA guide contained the SNV complementary base at position 6 and a synthetic mismatch at position 4, with a spacer length of 20 nt (highlighted with a red rectangle, Fig. 3B). We then evaluated whether the nucleotide selected as the artificial mismatch could improve the specificity of the SHERLOCK *CPSF3_(SNV)_* assay. We selected the best candidate obtained in the first screening (highlighted with a red rectangle, Fig. 3B), which used an uracil (U) as the mismatch (Fig. 4A), as well as two other new crRNAs, one using guanine (Fig. 4B) and the other cytosine (Fig. 4C) as the artificial mismatch. Using either an uracil or a guanine base as a mismatch, the SHERLOCK assays showed statistically significant differences in fluorescence intensity when comparing total RNAs from *CPSF3_(SNV)_* to WT RNA (Fig. 4A and C). Using a guanine, the fluorescence from the off-target (WT) sequence was reduced to near background (Fig. 4B). These data demonstrate that the SHERLOCK *CPSF3_(SNV)_* assay can be used to detect SNV in *T. brucei* cells resistant to acoziborole.

## DISCUSSION

Here, we describe the development of two SHERLOCK assays to screen for drug resistance in HAT, which could be used for epidemiological surveillance. There are currently few molecular diagnostics that can screen for emerging drug resistance in HAT patients, yet resistance to all the currently approved drugs used to treat HAT has been detected either in the field or can be generated in a laboratory. The two SHERLOCK assays described here can distinguish between WT and either gene chimeras or SNV targets. As an RNA diagnostic, these SHERLOCK assays can be used to screen patients presenting with relapse after treatment, which is critical for adapting drug treatment.

Although no longer used for the treatment of gHAT, melarsoprol is still recommended by the WHO for the treatment of rHAT at stage 2 in children or as a second option in adults. Unlike gHAT, rHAT is an acute infection, with disease progression taking from weeks to months. Resistance to melarsoprol would, therefore, represent a significant obstacle to rHAT control, particularly at a time when new cases of rHAT are still being reported (45) and when factors such as conflicts in endemic regions, climate change, and the dynamics of vectors and animal reservoirs continue to influence HAT epidemiology (46). In this study, we have developed a SHERLOCK assay targeting mutations in the *AQP2/3* locus, which were initially identified in circulating parasites by sequencing samples from patients with relapses after treatment (10, 11). Deletion and chimerization of the *TbAQP2* gene in several *T. b. gambiense* strains—and particularly the presence of the *AQP2/3_(814)_* chimera—have been associated with the lower sensitivity to melarsoprol and pentamidine (10, 30, 47). While our SHELROCK assays may help identify patients carrying drug-resistant strains or those at higher risk of relapse, it is not the sole determinant of treatment outcome (11, 48). This is illustrated by the case of the individual from whom the 348BT strain, which showed a high level of tolerance to melarsoprol, pentamidine, and suramin (11, 49), and contained the chimeric AQP2/3 variants described (11), but presented an efficient cure outcome after treatment with melarsoprol (Table 2). Nonetheless, the AQP2/3_(814)_-specific SHERLOCK assay has demonstrated the capacity to detect an unconfirmed drug-resistant strain (19BT) in a sample isolated from a patient successfully treated with eflornithine (19BT, Table 2). These findings support the potential of this tool as an aid in therapeutic decision-making for patients diagnosed in regions with a high incidence of treatment relapse. In total, our high-throughput AQP2/3_(814)_ SHERLOCK assay detected parasites involved in relapses without cross-reactivity and could be quickly implemented in reference labs in the field. One limitation is the intrinsic dependency of the method on prior knowledge of the gene mutations for the design of RPA primers and crRNAs. However, by using our existing SHERLOCK4HAT workflow (25), the assay can be rapidly adapted and implemented once a resistance-associated mutation has been identified.

Acoziborole will likely become the next frontline drug for the treatment of gHAT to move toward elimination of the disease by 2030 (20, 50). Like for the other drugs used to treat HAT, resistance to acoziborole can be generated in the laboratory through a single-point mutation in the *CPSF3* gene (22, 42). Several SHERLOCK assays have been developed to detect SNV (26, 28, 43), and *Lw*Cas13a, used in this study, is capable of tolerating mismatches, but with a resulting reduced RNA cleavage efficiency, and therefore reduced signal in a positive sample (51). This reduced sensitivity could be ameliorated by optimizing the crRNA guide length, the positioning of the mismatch, and/or the Cas13 variant used (43). In our *CPSF3_(SNV)_* SHERLOCK assay, we found that the most efficient combination was a 20 nt crRNA, the SNV at position 6, and the synthetic mismatch at position 4. Within the *CPSF3_(SNV)_* SHERLOCK assay, there is still scope for optimization to improve the specificity of the assay, and certain Cas13 variants could provide an even better discrimination *versus* potential off-targets. In our assays, the nucleotide selected for the mismatch had the most profound effect on the sensitivity of the assay, especially in the difference between on- and off-target. Here, the use of a guanine as the mismatch at position 4 showed the best discrimination between the two targets. Should resistance arise from an SNV or a chimeric gene formation, fully exploiting how the Cas13a-RNA complex is formed will be key to developing a robust assay for epidemiological surveillance.

SHERLOCK-based diagnostics serve as reactive tools to detect known antimicrobial resistance markers in field samples, but they are not predictive *per se* and require continuous updating with new crRNAs, as new resistance-associated mutations are regularly identified. While SHERLOCK can be rapidly adapted through either the addition of new crRNAs or multiplexing, PCR, PCR-RFLP, and targeted sequencing remain essential (11, 52, 53) for comprehensive genotyping and base-pair resolution of new alleles or mutations associated with resistance phenotypes. A combination of genotyping and SHERLOCK would provide a comprehensive workflow for passive surveillance and reactive detection of drug resistance for African Trypanosomiasis. This study demonstrates the versatility of the SHERLOCK technology to detect known genetic modifications directly associated with drug resistance, using existing workflows. We have shown that known resistance mechanisms, like through the formation of chimeras, are suitable targets for SHERLOCK. Given that for rHAT, where treatment with melarsoprol is still recommended in specific cases, the AQP2/3_(814)_ SHERLOCK assay could be readily used to screen for any relapse cases detected. Given the adaptability of SHERLOCK, generating a larger HAT antimicrobial resistance toolbox will be an invaluable asset for epidemiological surveillance to screen any cases of relapse. This will be critical not only to track the incidence of resistance but also for subsequent selection of the most individually adapted drug treatment.

## SUPPLEMENTAL MATERIAL

Supplemental figures aac.00933-25-s0001.pdf Figures S1 to S6.

Table S1 aac.00933-25-s0002.pdf RPA primers and crRNA templates used in this study.

ASM does not own the copyrights to Supplemental Material that may be linked to, or accessed through, an article. The authors have granted ASM a non-exclusive, world-wide license to publish the Supplemental Material files. Please contact the corresponding author directly for reuse.
