# Supplementary material for: A CRISPR-based diagnostic tool to survey drug resistance in human African trypanosomiasis
Source: Antimicrob Agents Chemother. 2025 Nov 18;69(12):e00933-25. doi: 10.1128/aac.00933-25 (PMC12691586; doi:10.1128/aac.00933-25)

Supplementary Figure 1. Key sequence alignments. (A) Alignments of the chimera *AQP2/AQP3*$_{(814)}$ (KF564931.1), WT *TbAQP2* (Tb927.10.14170) and *TbAQP3* (Tb927.10.14160) sequences. (B) Alignments of the WT *TbAQP2* (Tb927.10.14170) and *TbAQP3* (Tb927.10.14160); *AQP2/AQP3*$_{(814)}$ (KF564931.1); *AQP2/3*$_{(880)}$ (KM282050) and *AQP2/3*$_{(678\text{-}880)}$ (KM282034) sequences for the *AQP2/AQP3*$_{(814)}$-specific SHERLOCK design (C) or for the *TbAQP2*-specific SHERLOCK. Non-homologous regions are highlighted in gray, described SNPs are shown in red, and the protospacer flanking sequence of each crRNA is indicated in brackets. The sequences or the regions derived from the *TbAQP2* and *TbAQP3* genes are indicated in light and dark blue, respectively. The chimera breakpoint (position 814) is marked in the alignments where it is present.

Supplementary Figure 2. (A) crRNA screening for the detection of the AQP2/3$_{(814)}$ chimera by SHERLOCK using RNA from ChAQP2/3$_{(814)}$ cells and *Tbg* (*T. b. gambiense*) WT RNA as input. Statistical significance was assessed using Mann-Whitney U test. Asterisks indicate significance levels: $p<0.05$ (*) and (**) $p<0.01$. Violin-plots represent all the replicates by dots, the median, and the probability density distribution of the data (at least 3 replicates per conditions). (B) Limit of detection of *AQP2/3*$_{(814)}$-specific SHERLOCK (crRNA 2 and 3) using RNA extracted from ChAQP2/3$_{(814)}$ cells at concentrations ranging from 5 to 0.0025 ng/µL. The graph represents the mean ± standard deviation (SD), with individual replicate values represented as dots (at least 3 replicates per conditions).

Supplementary Figure 3. Validation of the *TbAQP2*-specific SHERLOCK assay. Validation of the SHERLOCK specific for the detection of the *TbAQP2* wild type, using RNA from *T.b.g.* WT, ChAQP2/3$_{(814)}$ and human embryonic kidney (HEK) cells, as well as using the ChAQP2/3$_{(814)}$ plasmid (10^5 c/µL). RNA inputs at 5 ng/µL. The graph represents the mean ±

standard deviation (SD), with individual replicate values represented as dots (at least 3 replicates per conditions).

Supplementary Figure 4. ROC analyses of the $AQP2/3_{(814)}$-specific SHERLOCK assays. Compilation of the positive (n=58) and negative (n=84) readouts obtained using the positive and negative controls throughout the optimization of the assay (left panels) and ROC curves (right panels) obtained for the analysis of each crRNA 2 (A) and 3 (B). The red dotted line marks the selected threshold for each test. (C) Summary of analytical features using each crRNA. Analytical sensitivity (95 % of CI), specificity (95 % of CI) and fold change cut-off established using the ROC analysis (thresholds of positivity, TH).

Supplementary Figure 5. RPA primer screening. Evaluation of long RPA primers pairs (30 nt) and shorter length primers (25 nt) by SHERLOCK assay using four potential crRNAs (1 to 4) specific for the detection of genetic material of $CPSF3_{(SNV)}$-edited cells. The data were represented as the fold-change over background fluorescence, taking the values obtained after 60 min of *Lw*Cas13a detection reaction. The graph represents the mean ± standard deviation (SD), with individual replicate (n=3) values represented as dots.

Supplementary Figure 6. Evaluation of the $AQP2/3_{(814)}$-specific SHERLOCK assays on isolates from HAT patients using the crRNA 3. (A) Representation of the fold-change over background fluorescence using the $APQ2/3_{(814)}$-specific SHERLOCK assay testing the different RNAs extracted from the 8 isolated *T. b. g.* strains 40AT, 130BT, 163AT, 349AT, 348BT, K03048, MBA and WT (Table 2). For all replicates an input concentration of 5 ng/µL was used. (B) Representation of the fold-change over background fluorescence using the $APQ2/3_{(814)}$-specific SHERLOCK assay testing the different RNAs extracted from patients in Mbuji-Mayi,

DRC (19BT, 48BT, 85BT, 93AT, 167BT, 167BT, 223AT, 340AT) and a WT strain. The input concentration used was conditioned by the availability of the sample in the range of 1.5 to 2.5 ng/µL. The graph represents the mean ± standard deviation (SD), with individual replicate values represented as dots (at least 3 replicates per conditions). The dotted lines show the thresholds of positivity established by ROC analysis for the crRNA 3 used in this study (Supplementary figure 4).

Supplementary Table 1. RPA primers and crRNA templates used in this study.

Supplementary figure 1

A

Sequence	Forward RPA Primer		crRNA 1 target region		crRNA 2 target region		crRNA 3 target region			Reverse RPA Primer	
**AQP2/AQP3** $_{(814)}$	**GTACGAAACGGTAGCTATTGGTGC**	[...]	**ATGGTCAACAACTTCGGCTTAGCGTCTC**	(C)	**CTTAGCGTCTCCCCTTGCGATGAATCCC**	(T)	**GCGTCTCCCCTTGCGATGAATCCCTCAC**	(T)	[...]	.814 **CTCTTCTTTTCTTTATGGTGGGGAGGTGT**	**841**
**WT.TbAQP2**	GTACGAAACGGTAGCTATTGGTGC	[...]	ATGGTCAACAACTTCGGCTTAGCGTCTC	(C)	CTTAGCGTCTCCCCTTGCGATGAATCCC	(T)	GCGTCTCCCCTTGCGATGAATCCCTCAC	(T)	[...]	CGGTGCGATCCTTCTCGGGGGGAAGTTC	841
WT.TbAQP3	ACACGAGCCGTTGGCAGTTGGTGC	[...]	ATTGGCAATAACATCGGTTACTCAACGG	(G)	TTACTCAACGGGTTACGCAATAAATCCG	(G)	TCAACGGGTTACGCAATAAATCCGGCTC	(G)	[...]	CTCTTCTTTTCTTTATGGTGGGAAGGTGT	817

B

Sequence	Forward RPA Primer		crRNA 2 target region	(PFS)	crRNA 3 target region	(PFS)		Reverse RPA Primer	
**AQP2/3(814)**	**GTACGAAACGGTAGCTATTGGTGC**	[...]	**CTTAGCGTCTCCCCTTGCGATGAATCCC**	(T)	**GCGTCTCCCCTTGCGATGAATCCCTCAC**	(T)	[...]	.814 **CTCTTCTTTTCTTTATGGTGGGGAGGTGT**	841
AQP2/3(880)	GTACGAAACGGTAGCTATTGGTGC	[...]	CTTAGCGTCTCCCCTTGCGATGAATCCC	(T)	GCGTCTCCCCTTGCGATGAATCCCTCAC	(T)	[...]	CGGTGCGATCCTTCTCGGGGGGAAGTTT	841
AQP2/3(678-880)	ACACGAGCCGTTGGCAGTTGGTGC	[...]	TTACTCAACGGGTTACGCAATAAATCCG	(G)	TCAACGGGTTACGCAATAAATCCGGCTC	(G)	[...]	CTCTTCTTTTCTTTATGGTGGGGAGGTGT	841
WT.TbAQP2	GTACGAAACGGTAGCTATTGGTGC	[...]	CTTAGCGTCTCCCCTTGCGATGAATCCC	(T)	GCGTCTCCCCTTGCGATGAATCCCTCAC	(T)	[...]	CGGTGCGATCCTTCTCGGGGGGAAGTTT	841
WT.TbAQP3	ACACGAGCCGTTGGCAGTTGGTGC	[...]	TTACTCAACGGGTTACGCAATAAATCCG	(G)	TCAACGGGTTACGCAATAAATCCGGCTC	(G)	[...]	CTCTTCTTTTCTTTATGGTGGGAAGGTGT	841

C

Sequence	Forward RPA Primer		crRNA target region	(PFS)		Reverse RPA Primer	
**WT.TbAQP2**	**GTACGAAACGGTAGCTATTGGTGC**	[...]	**GTCCCAGGGTCTTCGGTGCGATCCTTCT**	(T)	[...]	**GTTCCATTCTTTGGAGCTATCCTTG**	939
AQP2/3(678-880)	ACACGAGCCGTTGGCAGTTGGTGC	[...]	GTCCCAGGGTCTTCTCTTCTTTTCTTTA	(G)	[...]	GTTCCATTCTTTGGAGCTATCCTTG	939
AQP2/3(814)	GTACGAAACGGTAGCTATTGGTGC	[...]	GTCCCAGGGTCTTCTCTTCTTTTCTTTA	(G)	[...]	ATTCCATTGTTTGGAGGTATCTTTG	939
AQP2/3(880)	GTACGAAACGGTAGCTATTGGTGC	[...]	GTCCCAGGGTCTTCGGTGCGATCCTTCT	(T)	[...]	**A**TTCCATT**G**TTTGGAG**G**TATC**T**TTG	939
WT.TbAQP3	ACACGAGCCGTTGGCAGTTGGTGC	[...]	GTCCCAGGGTCTTCTCTTCTTTTCTTTA	(G)	[...]	ATTCCATTGTTTGGAGGTATCTTTG	915

Supplementary figure 2

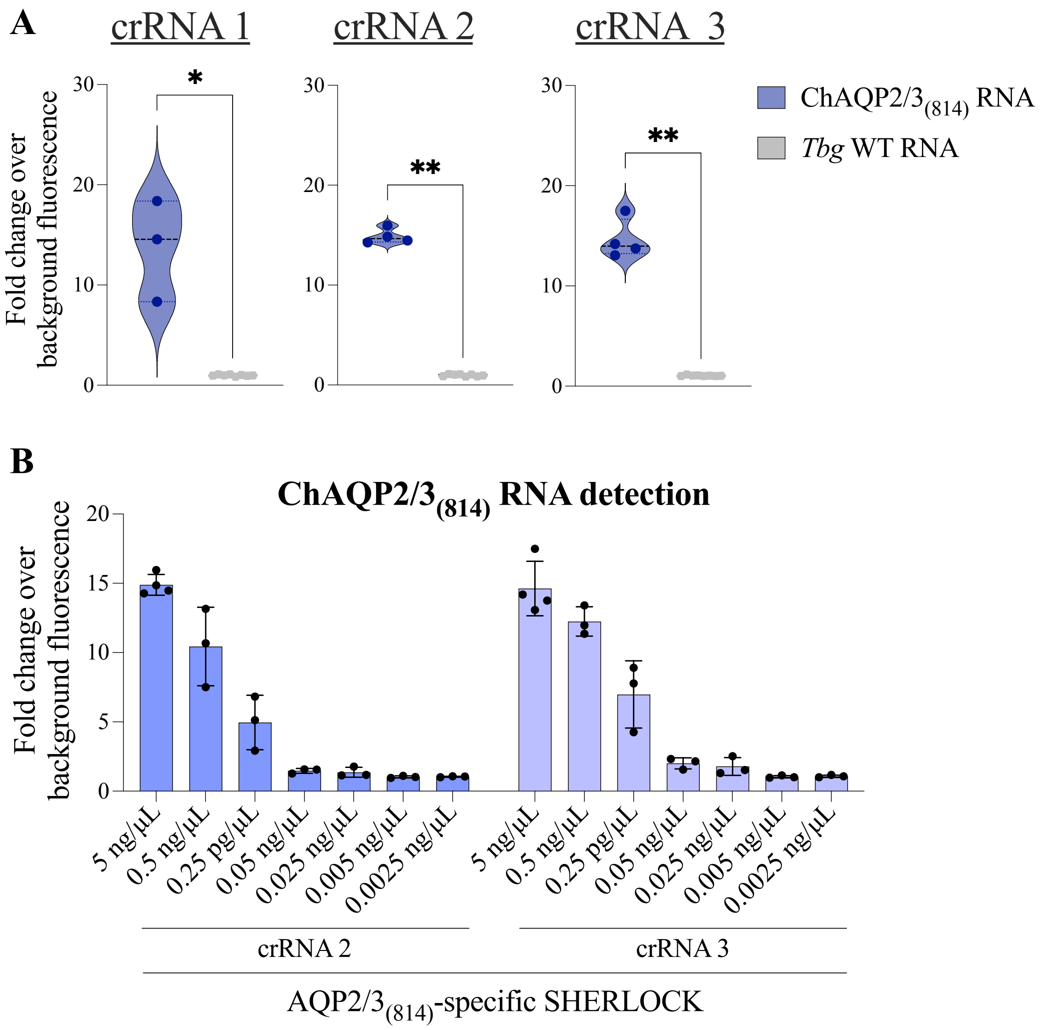

Supplementary figure 3

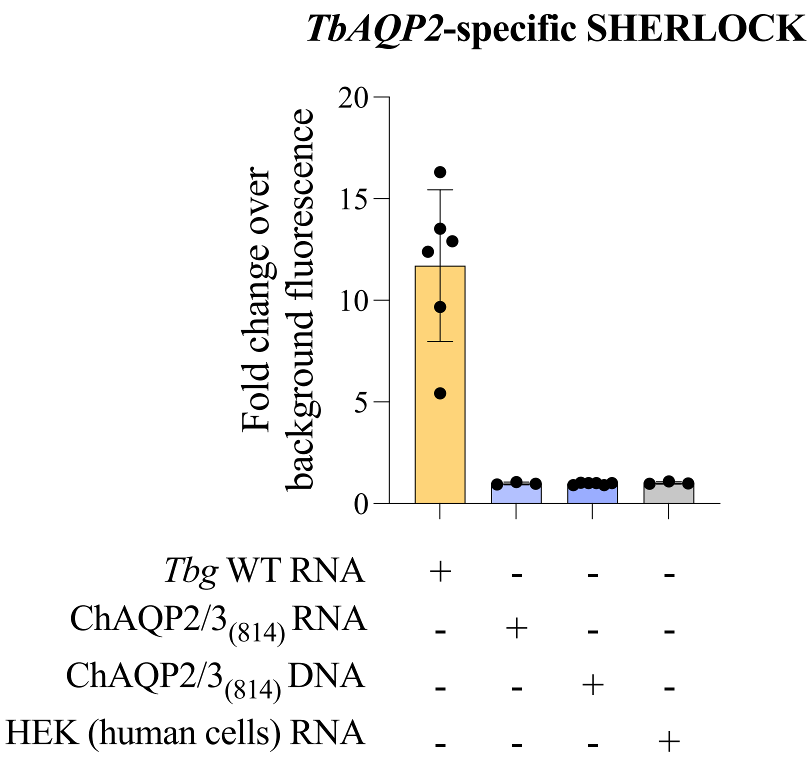

Supplementary figure 4

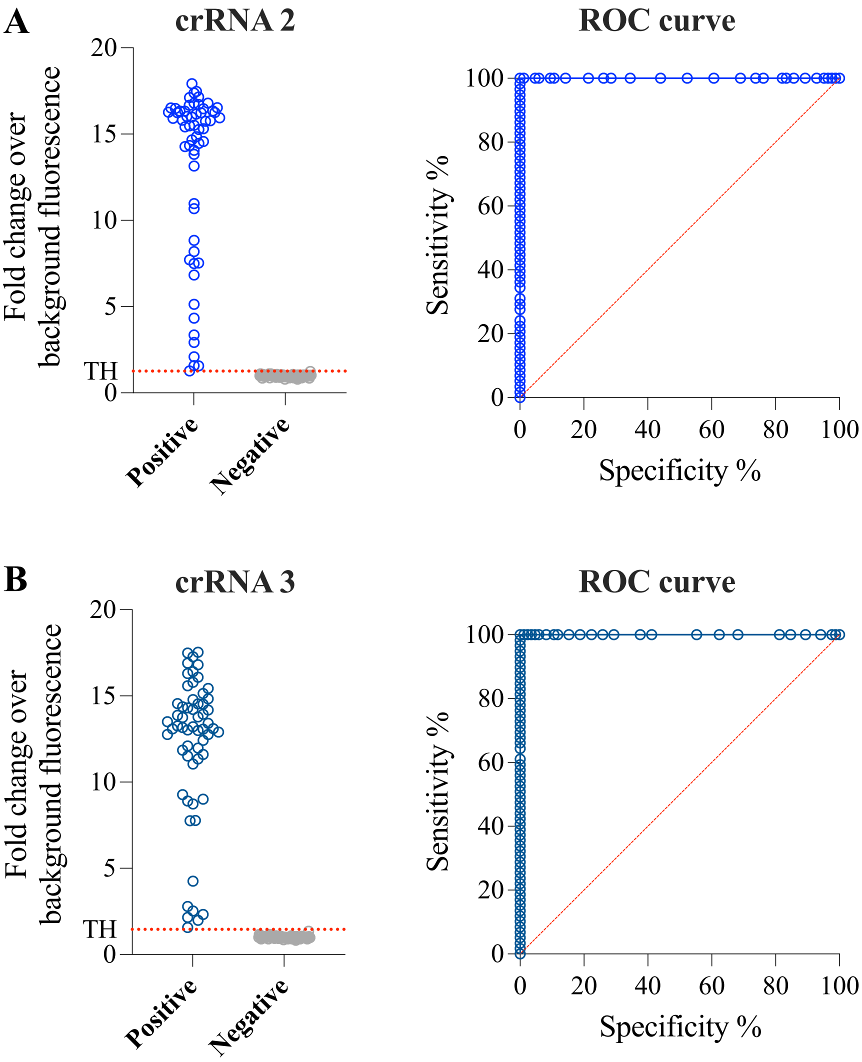

C

AQP2/3$_{(814)}$-specific SHERLOCK	**Sensitivity %** (95 % CI)	**Specificity %** (95% CI)	Fold-Change cutt-off (TH)
**crRNA 2**	**100.0** (93.79 to 100.0)	**100.0** (95.63 to 100.0)	> 1.260
**crRNA 3**	**100.0** (93.89 to 100.0)	**100.0** (95.68 to 100.0)	> 1.455

Supplementary Figure 5

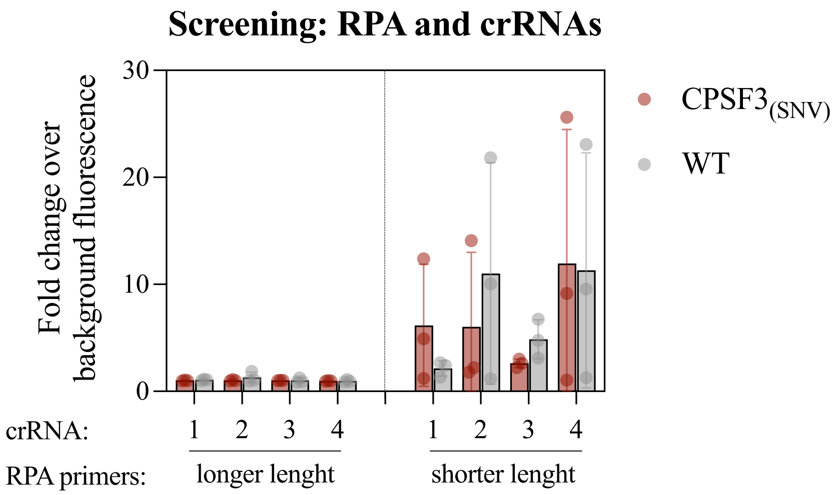

Supplementary figure 6

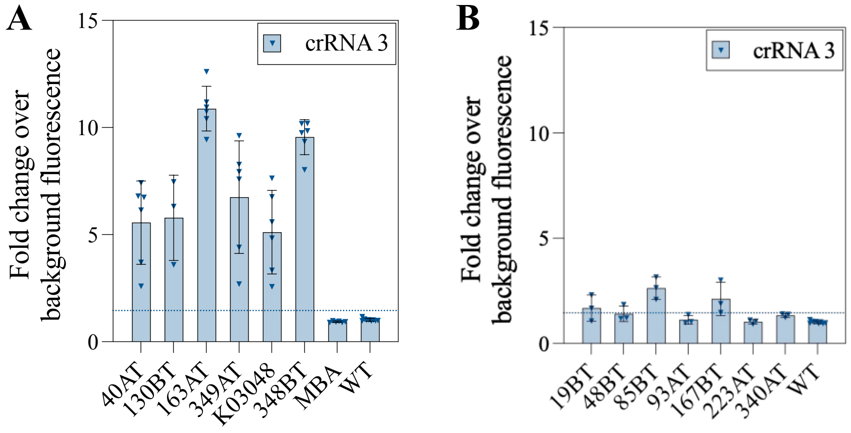

Supplement: Supplemental figures — Figures S1 to S6. [file aac.00933-25-s0001.pdf]
