# Supplementary material for: A CRISPR-based diagnostic tool to survey drug resistance in human African trypanosomiasis
Source: Antimicrob Agents Chemother. 2025 Nov 18;69(12):e00933-25. doi: 10.1128/aac.00933-25 (PMC12691586; doi:10.1128/aac.00933-25)
Supplement: Table S1 — RPA primers and crRNA templates used in this study. [file aac.00933-25-s0002.pdf]

Table S1. RPA primers and crRNA templates

		RPA forward primer with T7 promoter overhang 5'>3'			RPA reverse primer 5'>3'			crRNA DNA template (spacer + DR + T7 promoter) 5'>3'		
Target (accession number)	Amplicon (lenght)	name	sequence	region (lenght)	name	sequence	region (lenght)	name	sequence	region (lenght)
CPFS3 (XM_839191.1; Tb927.4.1340)	1 (99 nt)	CPSF3 Fw A	GAAATTAATACGACTCACTATAGGG CTTCTGGGAGCTGAAGTTCCACCGTACTCG	637 - 666 (30 nt)	CPSF3 RPA Rv A	GGATTCCCGCTCTTCACGTGACTCCAGTTC	735 - 706 (30 nt)	CPSF3 mut 1	GATATTCTCATTGCAGAGAGTACACATGTTTTAGTCCCCTTCGTTTTT<br>GGGGTAGTCTAAATCCCCTATAGTGAGTCGTATTAATTTC	670 - 696 (27 nt)
	2 (122 nt)	CPSF3 Fw B	GAAATTAATACGACTCACTATAGGG TGGGAGCTGAAGTTCCACCGTACTC	641 - 665 (25 nt)	CPSF3 RPA Rv B	GACATCGTGCACCCACGTCGTAAAC	762 - 738 (25 nt)	CPSF3 mut 2	ATTCTCATTGCAGAGAGTACACATGGT GTTTTAGTCCCCTTCGTTT<br>TTGGGGTAGTCTAAATCCCCTATAGTGAGTCGTATTAATTTC	673 - 699 (27 nt)
								CPSF3 SNVnt3 (A)	GATATTCTCATTGCAGAGAGTAGACATGTTTTAGTCCCCTTCGTTTTT<br>GGGGTAGTCTAAATCCCCTATAGTGAGTCGTATTAATTTC	670 - 696 (27 nt)
								CPSF3 SNVnt6 (B)	ATTCTCATTGCAGAGAGTACACAAGGT GTTTTAGTCCCCTTCGTT<br>TTTGGGGTAGTCTAAATCCCCTATAGTGAGTCGTATTAATTTC	673 - 699 (27 nt)
								CPSF3 SNVnt3 23nt (C)	TTCTCATTGCAGAGAGTAGACATGTTTTAGTCCCCTTCGTTTTTGGGG<br>TAGTCTAAATCCCCTATAGTGAGTCGTATTAATTTC	674 - 696 (23 nt)
								CPSF3 SNVnt3 20nt (D)	TCATTGCAGAGAGTAGACATGTTTTAGTCCCCTTCGTTTTTGGGGTAG<br>TCTAAATCCCCTATAGTGAGTCGTATTAATTTC	677 - 696 (20 nt)
								CPSF3 SNVnt6 23nt (E)	TCATTGCAGAGAGTACACAAGGT GTTTTAGTCCCCTTCGTTTTTGG<br>GGTAGTCTAAATCCCCTATAGTGAGTCGTATTAATTTC	677 - 699 (23 nt)
								CPSF3 SNVnt6 (mismatch A) 20nt (F)	TTGCAGAGAGTACACAAGGT GTTTTAGTCCCCTTCGTTTTTGGGGT<br>AGTCTAAATCCCCTATAGTGAGTCGTATTAATTTC	680 - 699 (20 nt)
								CPSF3 SNVnt6 (mismatch C) 20nt (F2)	TTGCAGAGAGTACACACGGT GTTTTAGTCCCCTTCGTTTTTGGGGT<br>AGTCTAAATCCCCTATAGTGAGTCGTATTAATTTC	680 - 699 (20 nt)
								CPSF3 SNVnt6 (mismatch G) 20nt (F3)	TTGCAGAGAGTACACAGGGT GTTTTAGTCCCCTTCGTTTTTGGGGT<br>AGTCTAAATCCCCTATAGTGAGTCGTATTAATTTC	680 - 699 (20 nt)
								CPSF3 contiguos msmtch SNVnt3 23nt (G)	TTCTCATTGCAGAGAGTACTCATGTTTTAGTCCCCTTCGTTTTTGGGG<br>TAGTCTAAATCCCCTATAGTGAGTCGTATTAATTTC	674 - 696 (23 nt)
								CPSF3 contiguos msmtch SNVnt3 20nt (H)	TCATTGCAGAGAGTACTCATGTTTTAGTCCCCTTCGTTTTTGGGGTAG<br>TCTAAATCCCCTATAGTGAGTCGTATTAATTTC	677 - 696 (20 nt)
								CPSF3 contiguos msmtch SNVnt6 23nt (I)	TCATTGCAGAGAGTACACTTGGTGTTTTAGTCCCCTTCGTTTTTGGGG<br>TAGTCTAAATCCCCTATAGTGAGTCGTATTAATTTC	677 - 699 (23 nt)
								CPSF3 contiguos msmtch SNVnt6 20nt (J)	TTGCAGAGAGTACACTTGGTGTTTTAGTCCCCTTCGTTTTTGGGGTAG<br>TCTAAATCCCCTATAGTGAGTCGTATTAATTTC	680 - 699 (20 nt)
								CPSF3 WT 1	GATATTCTCATTGCAGAGAGTACAAATGTTTTAGTCCCCTTCGTTTTT<br>GGGGTAGTCTAAATCCCCTATAGTGAGTCGTATTAATTTC	670 - 696 (27 nt)
								CPSF3 WT 2	ATTCTCATTGCAGAGAGTACAAATGGT GTTTTAGTCCCCTTCGTTT<br>TTGGGGTAGTCTAAATCCCCTATAGTGAGTCGTATTAATTTC	673 - 699 (27 nt)
T. brucei gambiense AQP2/AQP3 $_{(814)}$ chimera (KF564935.1)	1 (137 nt)	chimera AQP2/AQP3 Fw 1	GAAATTAATACGACTCACTATAGGG GTACGAAACGGTAGCTATTGGTGC	705 - 728 (24 nt)	chimera AQP2/AQP3 Rv 1	ACACCTCCCCACCATAAAGAAAAGAAGAG	841 - 813 (29 nt)	chimera AQP2/AQP3 1	ATGGTCAACAACTTCGGCTTAGCGTCTCGTTTTAGTCCCCTTCGT<br>TTTTGGGGTAGTCTAAATCCCCTATAGTGAGTCGTATTAATTTC	742 - 769 (28 nt)
	2 (100 nt)	chimera AQP2/AQP3 Fw 2	GAAATTAATACGACTCACTATAGGG CTTGTCTTCGTCATGGTCAACAACTT	730 - 755 (25 nt)	chimera AQP2/AQP3 Rv 2	CATAAAGAAAAGAAGAGAAGACCCTGG	829-803 (25 nt)	chimera AQP2/AQP3 2	CTTAGCGTCTCCCCTTGCGATGAATCCCGTTTTAGTCCCCTTCGT<br>TTTTGGGGTAGTCTAAATCCCCTATAGTGAGTCGTATTAATTTC	759 - 786 (28 nt)
								chimera AQP2/AQP3 3	GCGTCTCCCCTTGCGATGAATCCCTCACGTTTTAGTCCCCTTCGT<br>TTTTGGGGTAGTCTAAATCCCCTATAGTGAGTCGTATTAATTTC	763 - 790 (28 nt)
T. brucei gambiense AQP2 (KM282049.1)					AQP2 Rv 3	CAAGGATAGCTCCAAAGAATGGAAC	880 - 904 (25 nt)	AQP2 A	TCGGTGCGATCCTTCTCGGGGGGGAAGT GTTTTAGTCCCCTTCG<br>TTTTTGGGGTAGTCTAAATCCCCTATAGTGAGTCGTATTAATTTC	812 - 839 (28 nt)
								AQP2 B	TCTTCGGTGCGATCCTTCTCGGGGGGGA GTTTTAGTCCCCTTCG<br>TTTTTGGGGTAGTCTAAATCCCCTATAGTGAGTCGTATTAATTTC	809 - 836 (28 nt)
								AQP2 C	GTCCCAGGGTCTTCGGTGCGATCCTTCTGTTTTAGTCCCCTTCGT<br>TTTTGGGGTAGTCTAAATCCCCTATAGTGAGTCGTATTAATTTC	800 - 827 (28 nt)
